# Experimental investigation of thermal performance of vertical multitube cylindrical latent heat thermal energy storage systems

**DOI:** 10.1007/s11356-024-31864-7

**Published:** 2024-01-08

**Authors:** Gang Shen, Xiaolin Wang, Jie Yu, Yuejing Bin, Shan Zhong, Shengqi Yang, Jianguo Wang

**Affiliations:** 1 School of Mechanical and Resource Engineering, Wuzhou University, Wuzhou, 543002 China; 2 School of Engineering, University of Tasmania, Hobart, TAS 7001 Australia; 3 School of Mechanics and Civil Engineering, China University of Mining Technology, Xuzhou, China

**Keywords:** Latent heat storage, Thermal performance, Phase change material, Natural convection, Shell-and-tube heat exchanger, Multiple tube

## Abstract

The multitube design in the shell-and-tube type latent heat thermal energy storage (LHTES) system has received intensive attention due to its promising benefits in enhancing heat storage efficiency. In this paper, single and multi-tube shell LHTES systems were experimentally investigated. First, this study experimentally compared the thermal characteristics between a multiple-tube heat exchanger (MTHX) and a single-tube heat exchanger (STHX). The STHX’s geometrical parameters coincided with a virtual cylindrical domain in the MTHX, being similar to the single-tube model formulated by simplifying the numerical solution to investigate the MTHX. The experimental data was then used to validate the simplified numerical model commonly used in the literature that converted the multi-tube problem to a single-tube model by formulating a virtual cylindrical domain for each tube in the MTHX system. The results showed that there was a noticeable difference in the thermal characteristics between the actual STHX and the virtual cylindrical STHX domain in the MTHX system. The comparison indicated that the simplified numerical model could not accurately reflect the thermal performance of the MTHX system. An experimental study or three-dimensional numerical modelling was required for the thermal analysis of the multi-tube problems. Second, the effect of tube number in the MTHX was experimentally investigated. It was found that an increase in tube number boosted both charging and discharging rates without inhibiting the natural convection. The five-tube configuration decreased the total charging and discharging duration by 50% compared to the two-tube one. Finally, the effect of heat transfer fluid (HTF) operating parameters on the system performance was evaluated on the five-tube MTHX system. The results revealed that the adoption of higher HTF temperature considerably improved the charging performance. The charging time decreased by up to 41% with the HTF temperature increasing from 70 to 80 °C. Meanwhile, a variation in the HTF flow rate from 5 to 20 L/min showed a more pronounced influence on charging than on discharging due to the different dominant heat transfer mechanisms.

## Introduction

Thermal energy storage plays an important role in solar energy applications to boost the efficiency of energy utilization in heating and cooling. Latent heat thermal energy storage (LHTES) is one of the most attractive storage technologies due to its large energy storage density and capacity. Different organic and inorganic phase change materials (PCMs) are developed and applied in the LHTES system to meet the various demands in industrial, commercial, and residential sectors. However, the low thermal conductivity of PCMs lowers the efficiency of an LHTES system thus restricting its usability and large-scale deployment. Over the last decades, significant research efforts have been committed to improving the performance of LHTES systems in energy storage and retrieval (Al-Maghalseh and Mahkamov 2018; Mishra et al. 2022; Naveenkumar et al. 2022). The cylindrical LHTES system is the most popularly studied storage technology (Kalapala and Devanuri 2018).

Several techniques were proposed to enhance the thermal performance of the cylindrical LHTES systems including (1) system mounting orientation such as vertical, horizontal, or inclined with specific angles (Seddegh et al. 2016; Kousha et al. 2017); (2) PCM and heat transfer fluid (HTF) tube arrangements, e.g., cylinder mode (Han et al. 2017), and the eccentrically located HTF tube (Yusuf Yazıcı et al. 2014; Zheng et al. 2018; Modi et al. 2022); (3) optimization of design and operating parameters, e.g., optimal and suitable radius ratio (Seddegh et al. 2017; Shen et al. 2020; Modi et al. 2023), optimal shell tilting angle (Seddegh et al. 2018; Shen et al. 2019b), and the optimized design and operating parameters (Dhanapal et al. 2022); and (4) extension in heat transfer surface area including finned-tube (Rathod and Banerjee 2015) and multiple tubes (Joybari et al. 2019; Kousha et al. 2019; Shen et al. 2019a). As a promising configuration in the case of a large cylindrical LHTES system, the multiple-tube design is efficient in increasing the heat transfer area between the PCM and HTF and enhancing PCM natural convection (Agyenim et al. 2010). Furthermore, the multiple-tube design offers more flexibility in arranging the HTF flow (Pizzolato et al. 2019), employs different types of HTFs, or simultaneously operates the HTF with distinct conditions in temperature and flow rate (Murray and Groulx 2014a, b).

Agyenim et al. (2010) experimentally compared the melting performance of two configurations in a horizontal cylindrical LHTES system, one with a single HTF tube and another with four HTF tubes. It was revealed that the separate flow cells formed from multiple tubes led to increased convection heat transfer, hence a reduced melting time. Further, Agyenim (2016) investigated three enhancement techniques applied to a horizontal shell-and-tube LHS unit built in the lab, the multi-tube, the longitudinal-fin tube, and the circular-fin tube. It was reported that the multitube showed superior performance over the others in terms of the total melting time and overall utilization efficiency.

With the numerical studies, Esapour et al. (2016a, b) investigated a series of horizontal cylindrical LHS systems and found that the multitube design effectively increased heat flux, reducing the melting time by up to 29%. Besides, given the same tube number, the lower locations of HTF tubes were advantageous to the melting performance compared to the other arrangements. Niyas et al. (2017a) numerically examined the effect of tube numbers in a set of horizontal multi-tube LHTES prototypes with a constant PCM amount. They found that an addition in tube number reduced the discharging time, but the enhancement effect turned out to be marginal with the tube number exceeding 25. Furthermore, based on the optimal design obtained from the previous numerical study, Niyas et al. (2017b) built a horizontal multi-tube LHS experimental rig. It was reported that the charging process observed more significant variation in PCM temperature along the angular direction than the discharging, which was attributed to the different roles of natural convection on the two processes. Later Esapour et al. (2018) developed a horizontal multiple-tube heat exchanger model with metal foam embedded. The numerical results showed the inner tube arrangement exhibited a higher enhancement rate on charging than on discharging.

With the lattice Boltzmann simulation, Luo et al. (2015) found that the centrosymmetric allocation of the tubes demonstrated better melting performance compared to inline and staggered arrangements with the same inner tube number configured in a horizontal LHTES unit. Kousha et al. (2019) experimentally studied a set of horizontal multi-tube LHTES units with RT35 used as the storage medium. The team reported that a rise in tube number simultaneously boosted the melting and solidification rates, while the enhancement magnitude depended on both the tube number and distribution.

Several researchers presented the impact of fins and HTF operating conditions on the performance of multitube LHTES systems. An experimental investigation by Anish et al. (2019) studied the effect of various HTF parameters in a horizontal multi-finned-tube LHTES system. It was found that the HTF inlet temperature exhibited a more significant role than its flow rate on the average temperature and storage effectiveness. Dandotiya and Banker (2017) numerically analyzed the influence of fin placements on the thermal behavior of a horizontal multitube heat exchanger. Bhagat et al. (2018) optimized the fin design in a vertical multi-tube LHTES unit to minimize the fluctuation in HTF outlet temperature using a three-dimensional numerical model. They reported that the number and thickness of fins were more important than the material of fins to enhance the heat transfer between PCM and HTF. Abreha et al. (2020) modelled an LHTES system with multiple finned tubes embedded in the horizontal shell. The numerical study analyzed important performance indicators including stored energy, liquid fraction, and average temperature. It was concluded that the design along with the chosen PCM and HTF was a promising LHTES application.

The literature review indicated that multitube design could effectively advance the heat transfer performance of a horizontal multitube LHTES system with its inner tube configuration has been widely investigated. Due to the presence of natural convection and multiple heat sources, the vertical multiple-tube heat exchanger (MTHX) and the horizontal one feature distinctly different heat transfer mechanisms. However, limited experimental and numerical efforts have been undertaken on the vertical MTHX to reveal its thermal characteristics. The tube arrangement in the vertical MTHX significantly influences the phase change process, and its effect on natural convection flow needs further in-depth investigation. Moreover, the numerical modelling of the vertical MTHX still commonly relies on a single-tube model formulated from a virtual cylindrical domain concentric with each tube (Trp et al. 2006; Pirasaci and Goswami 2016; Tehrani et al. 2016; Fang et al. 2018). Although the validity of the simplifying solution has been questioned by a few studies (Agyenim et al. 2010; Joybari et al. 2019; Pizzolato et al. 2019), not much further work has been carried out. An experimental comparison study between an MTHX and a single-tube heat exchanger (STHX) coinciding with the simplifying solution is necessary. Another limitation of the simplifying single-tube model is being unable to deal with the effects of inner tube number and allocation within a vertical MTHX, which is critical to designing an efficient LHTES system. To address these research gaps, this study first experimentally compares the thermal characteristics of an STHX and a five-tube MTHX during a complete charging and discharging cycle. The STHX’s geometrical parameters coincide with the virtual PCM cylindrical boundary built within the MTHX for simplifying the multitube model. In this way, the comparative investigation of the STHX and MTHX explains to what extent the simplifying approach is valid. The second study objective is to experimentally investigate the effect of the inner tube numbers in the vertical MTHXs. This is conducted by implementing two- and four-inner-tube HXs with the same storage container and PCM amount as the five-tube MTHX and comparing the thermal responses of these three HXs. Finally, the effect of HTF operating parameters is experimentally investigated on the five-tube MTHX. This study presents valuable insights and guidelines for the multitube design to maximize an LHTES system’s thermal performance.

## Experimental setup

### Experimental rig and procedure

**Fig. 1:**
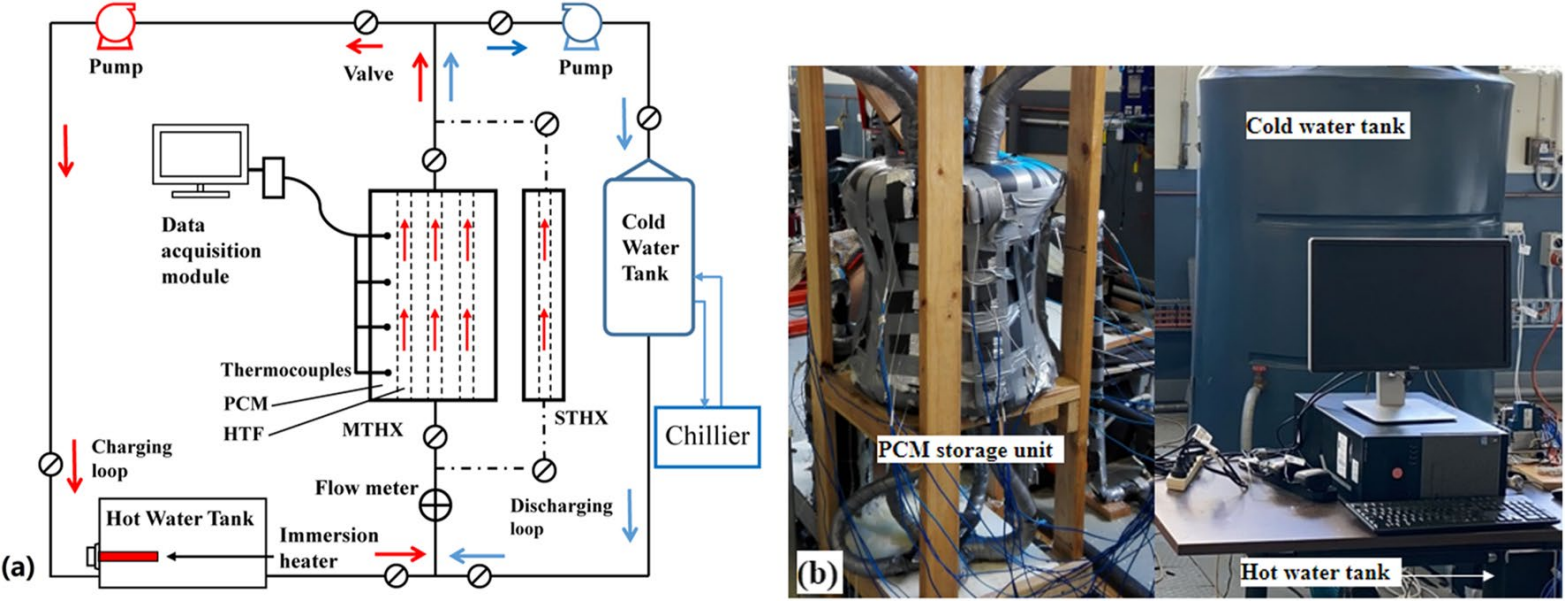
Schematic diagram (**a**) and photo (**b**) of the experimental system

Figure 1 shows the schematic diagram and photo of the experimental system, which consists of vertical shell-and-tube HXs, hot and cold water tanks, pumps and valves. These components were fitted with copper tubes to form charging and discharging loops for water to circulate through as the HTF. An ultrasonic liquid flow meter (SICK FFUS15-1G1IO, analog output, maximum flow ≤ 36 L/min) with an accuracy of ± 2% was installed at the inlet pipe of the HX to measure and monitor the hot/cold HTF flow rate. Type T thermocouples with an accuracy of ± 0.2 °C were used to measure PCM temperature as well as HTF inlet and outlet temperatures. Thermocouple and flow meter cables were connected to the desktop-computer-based data loggers (National Instruments NI9411). The software package LabVIEW was used to acquire data from the data loggers and save the measured temperatures and flow rates at a time interval of 5 s (data saved to CSV file).

Two 2.4 kW electric immersion heaters with precise temperature control were installed in the hot water tank to maintain the water at the required temperature. A 20 kW chiller maintained the HTF at 15 °C in the cold water tank with a volume capacity of 1600 L. During charging, the hot HTF was driven by a vertical centrifugal pump to flow upward through the vertical LHTES unit to melt the PCM and then flow back to the hot water tank. Immediately following the end of charging, a discharging process was launched to solidify the PCM with a horizontal centrifugal pump which channels the cold HTF upward through the LHTES unit. Before each test, the cold HTF of 15 °C was circulated through the LHS unit, having all thermocouple readings stabilized to close to the HTF inlet temperature.

**Fig. 2:**
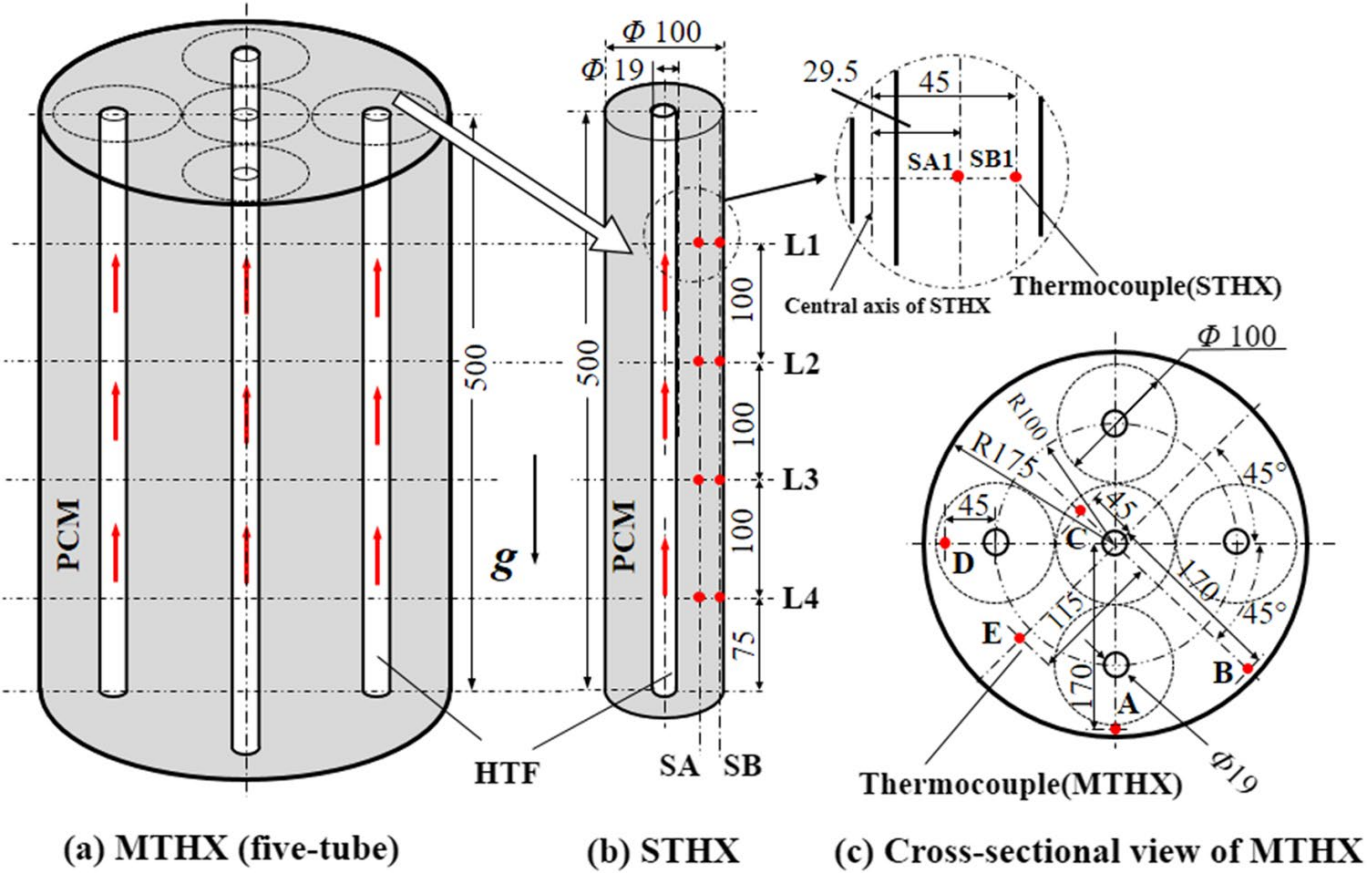
The schematic of the five-tube MTHX (**a**), the thermocouple positions within the STHX (**b**), and the cross-sectional view of the MTHX (**c**) (Unit: mm)

Figure 2 presents the sketches of the MTHX and STHX employed as the LHTES units in the study, which were manufactured with the same height of 500 mm. The inner tube spacing in the MTHX was chosen such that an optimal shell-to-tube radius ratio of 5.4 obtained by an earlier experimental investigation (Seddegh et al. 2017) was applied to each virtual boundary (shown by dotted lines around each tube in Fig. 2a and c). The STHX was built with a shell inner diameter of 100 mm and with the same inner tube as those in the MTHX, matching exactly with the virtual PCM cylindrical boundary surrounding each tube in the MTHX. Both heat exchanger containers were made of transparent polypropylene with a thickness of 6 mm and a thermal conductivity of 0.11 W/(m·K), insulated with Armaflex foam sheets with a thickness of 20 mm and a thermal conductivity of 0.036 W/(m·K). In both heat exchangers, the PCM was stored in the annulus between the outer shell and inner tube(s) while the HTF was forced to flow through the tube(s).

**Fig. 3:**
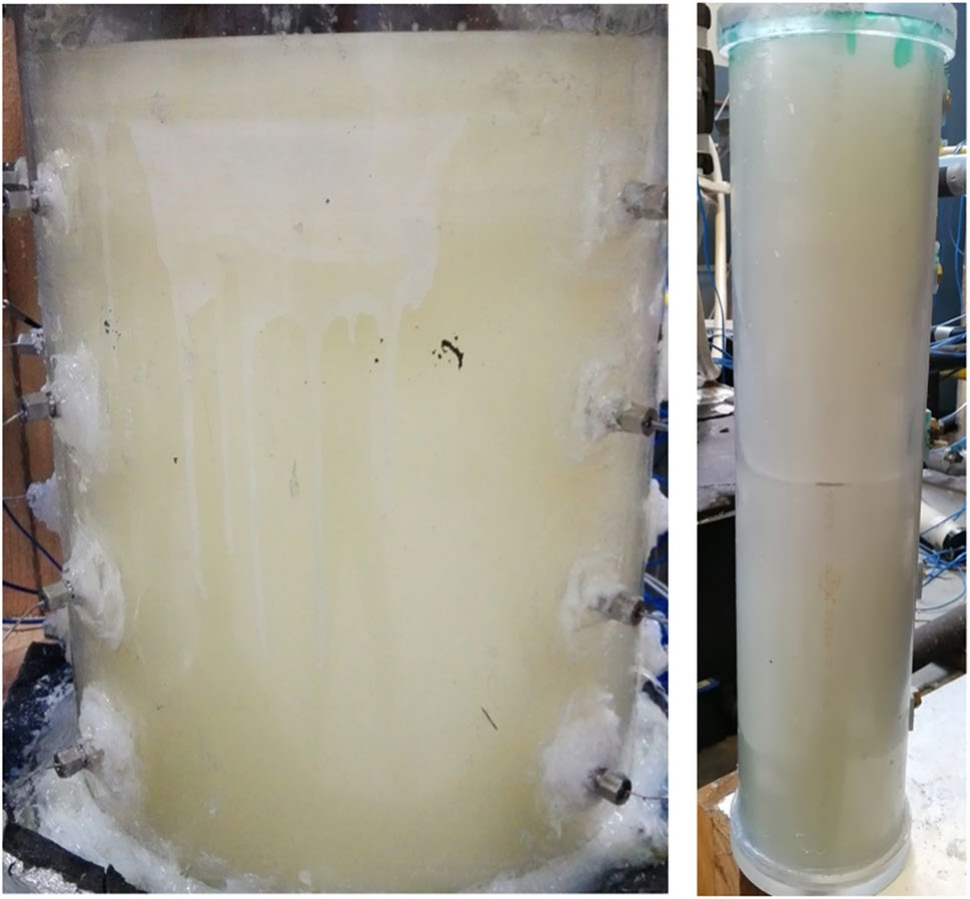
Photographs of the MTHX (left) and STHX (right)

To compare the PCM solid–liquid phase change process, all thermocouples within the STHX and MTHX were placed at the same four height levels as shown in Fig. 2b. A set of thermocouples with the same radial location and at the different height levels was grouped as position A, B, C, D, or E within the MTHX, and SA, or SB within the STHX. SA and SB were fixed at respective radial distances of 29.5 and 45 mm from the STHX tube central axis (Fig. 2b). Position SA is to monitor the melting front between the liquid and solid PCM during the melting and solidification process. Position SB is about 5 mm from the shell’s inner surface to monitor if all PCM melts at the corresponding level and also ensure the minimum impact from surroundings. Five positions A, B, C, D, and E are presented in a cross-sectional view of the five-tube HX (Fig. 2c). Positions A and B in the MTHX were mounted at the same radial distance of 5 mm from the shell inner surface while with different angular positions to monitor the PCM status. These two positions could allow the comparison of the impact of the HTF on the PCM melting. It also allows monitoring if all PCM melts at this corresponding level. Positions C and D were placed 45 mm away from the central axes of central and outer tubes, reflecting the performance of central and outer tubes, respectively. As positions C and D in the MTHX are located at the same radial distance from the tubes as position SB in the STHX, they were comparable to reveal the deviation in thermal performance between the STHX and the central and outer virtual cylindrical boundaries in the MTHX. The comparison between these positions could be a way to verify the simplifying numerical approach in the modelling of the MTHX. Finally, position E in the MTHX was located 115 mm away from the container’s central axis, intending to monitor the combined effect from the adjacent tubes in the MTHX. Table 1 lists the key geometrical parameters of the studied STHX and MTHXs. Figure 3 presents the photographs of the STHX and five-tube MTHX.

**Table 1:** Key geometrical parameters of the STHX and the MTHXs

LHS unit	Tube number	Height	Tube outside diameter	Shell inner diameter	PCM mass
	(-)	*H* (mm)	*d* (mm)	*D* (mm)	*m* (kg)
STHX	1	500	19	100	2.9
MTHX	5	500	19	350	35.0
	4				
	2

**Fig. 4:**
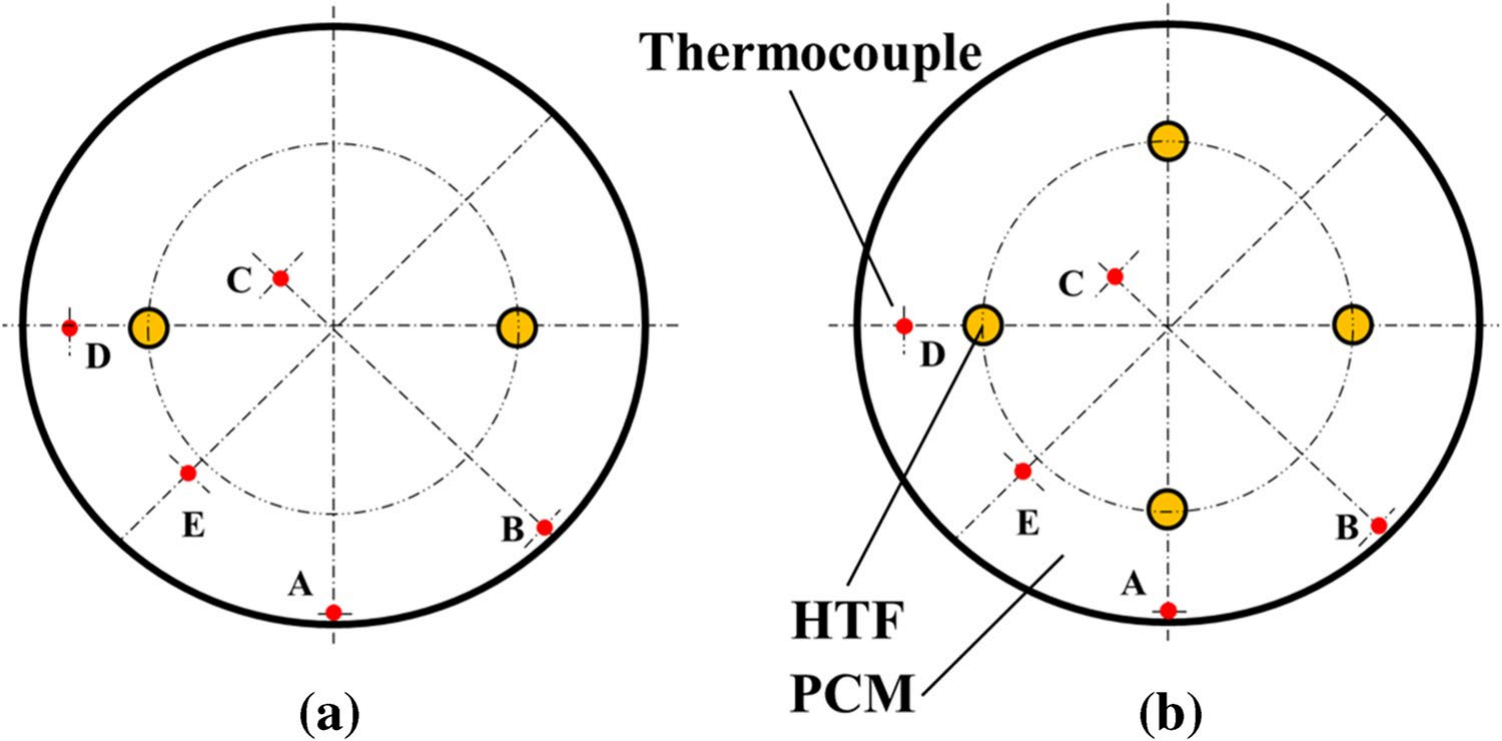
Cross-sectional views of a two- and b four-inner-tube in the MTHXs

In the case of varying the inner tube number in the MTHXs, Fig. 4 shows the cross-sectional view of the two- and four-tube HXs. The thermocouple positions, tube dimensions, and the tube radial distance from the container central axis in the two- and four-tube HXs remain the same as those in the five-tube one. By varying the tube number, the total volume flow rate was fixed at 20 L/min and evenly distributed among the inner tubes. The selection of this flow rate is based on the hot water flow rate required in the household hot water system in the US and Australia. Table 2 summarizes the operating parameters for the studied STHX and MTHXs.

**Table 2:** Studied operating parameters in the STHX and MTHXs

LHS unit	Tube number	HTF charging temperature	HTF discharging temperature	Total volume flow rate	Reynolds number per tube
	(-)	*T*_ch_ (°C)	*T*_dis_ (°C)	*V* (L/min)	Re (-)
STHX	1	80	15	4	14,000
MTHX	5	85, 80, 75, 70	15	20/5	14,000/3500
	4	80		20	17,500
	2	80		20	35,000

### PCM properties

Previous studies (Shukla et al. 2009; Seddegh et al. 2015) revealed the PCM with a phase change temperature range between 50 and 70 °C is advantageous to low-temperature heat storage applications with solar collector assisted. Given the additional factors that considered including cost and reliability, the present study chose paraffin wax RT60 as the heat storage medium, produced by Rubitherm, Gmbh. Table 3 lists the thermophysical properties of the PCM.

**Table 3:** Thermophysical properties of RT 60 (Rubitherm Technologies GmbH 2022)

Properties	Value
Phase transition temperature range	55–61 °C (*T*_solidus_ -*T*_liquidus_)
Density	880 kg/m^3^ (15 °C),770 kg/m^3^ (80 °C)
Thermal conductivity, liquid/solid	0.2 W/(m·K)
Thermal expansion coefficient of liquid phase	0.76E-3 /K
Dynamic viscosity	3.08E-3 kg/(m·s)
Specific heat, liquid/solid	2 kJ/(kg·K)
Latent heat of fusion	130 kJ/kg

## Experimental data reduction and heat loss analysis

### Experimental data reduction

The charging and discharging times, the average temperature, and total stored energy were utilized to evaluate the performance of the LHTES systems. The charging or discharging time was calculated given that all thermocouple readings in an HX did not change noticeably. Following an approach reported in the literature (Caron-Soupart et al. 2016; Seddegh et al. 2017), the present work applied the weighting method to calculate the average temperature and total stored energy for each HX with the thermocouples’ recordings available.

The weighting factor *ω*_i_ assigned to the reading of a specific thermocouple is defined as the ratio of the volume of the PCM element surrounding the thermocouple to the total PCM volume in an HX. As described in the “Experimental rig and procedure” section, the total PCM amount and thermocouple positions differed greatly between the STHX and MTHX. As a result, each thermocouple had a different PCM volume surrounded depending on its location in the heat exchanger. Therefore, the weighting method was able to take into account the difference in the PCM element volume of each thermocouple it corresponds to. The average temperature is given by

1 $$\overline{T} = \sum\limits_{{{\text{i}} = 1}}^{{\text{n}}} {T_{{\text{i}}} \omega_{{\text{i}}} }$$

where *T*_i_ is the measured temperature assigned to the element surrounding the thermocouple, and *n* is the total number of elements (thermocouples). The liquid fraction in each element is calculated as follows:

2 $${\phi }_{{\text{i}}}=\left\{\begin{array}{lc}0,& {T}_{{\text{i}}}\le {T}_{{\text{solidus}}}\\ \frac{{T}_{{\text{i}}}-{T}_{{\text{solidus}}}}{{T}_{{\text{liquidos}}}-{T}_{{\text{solidus}}}},{T}_{{\text{solidus}}}& <{T}_{{\text{i}}}<{T}_{{\text{liquidos}}}\\ 1,& {T}_{{\text{i}}}\ge {T}_{{\text{liquidos}}}\end{array}\right.$$

where *T*_solidus_ and *T*_liquidus_ are the PCM solidus and liquidus temperatures, respectively. With the local liquid fraction attained, the stored heat *Q*_i_ within each element, and the total stored heat *Q* by the HX would be

3 $$Q_{{\text{i}}} = \left\{ {\begin{array}{*{20}l} {m_{{\text{i}}} C_{p} (T_{{\text{i}}} - T_{{{\text{ini}}}} ),} \hfill & {T_{{\text{i}}} \le T_{{{\text{solidus}}}} } \hfill \\ {m_{{\text{i}}} C_{p} (T_{{\text{i}}} - T_{{{\text{ini}}}} ) + m_{{\text{i}}} \phi_{{\text{i}}} L,} \hfill & {T_{{{\text{solidus}}}} < T_{{\text{i}}} < T_{{{\text{liquidus}}}} } \hfill \\ {m_{{\text{i}}} C_{p} (T_{{\text{i}}} - T_{{{\text{ini}}}} ) + m_{{\text{i}}} \phi_{{\text{i}}} L + m_{{\text{i}}} C_{p} (T_{{\text{i}}} - T_{{{\text{liquidus}}}} ),} \hfill & {T_{{\text{i}}} \ge T_{{{\text{liquidus}}}} } \hfill \\ \end{array} } \right.$$

4 $$Q = \sum\limits_{{\text{i}}}^{{\text{n}}} {\omega_{{\text{i}}} Q_{{\text{i}}} }$$

where *T*_ini_ is the initial temperature of PCM for both charging and discharging cycles, *m*_i_ is the mass of the PCM element surrounding the thermocouple, and *L* is the latent heat of fusion. It can be deduced from Eqs. 3 and 4 that within a complete charging and discharging cycle, the total stored heat *Q* increased from zero at the initial test state to a maximum value at the end of charging and then dropped back to zero at the end of discharge.

### Heat loss analysis

As noted in the “Experimental rig and procedure” section, the PCM storage enclosures used in the study were designed and built to minimize the heat loss caused by the temperature difference between the storage tank and its surroundings. The rate of heat loss to the environment is estimated as (Erek and Dincer 2008; Ezan et al. 2011)

5 $$\mathop Q\limits^{ \bullet }_{{{\text{loss}}}} (t) = \widetilde{U}A(\overline{T}_{{{\text{inner}}}} - \overline{T}_{{{\text{outer}}}} )$$

where $\widetilde{U}$ stands for the overall heat transfer coefficient, which is assumed to be equal between the STHX and the MTHX given the same specifications of the enclosure and the insulation configured. *A* is the outer surface area of an LHTES unit, while $\overline{T}_{{{\text{outer}}}}$ is the average temperature of the environment recorded in the laboratory (~ 19 °C). $\overline{T}_{{{\text{inner}}}}$ is the temperature of the PCM layer adjacent to the container’s inner surface, evaluated with the average recordings of position SB in the STHX or positions A and B in the MTHX. The maximum heat loss rate of the STHX with the charging temperature of 80 °C operated is approximately 10 W which is consistent with that reported in the literature (Seddegh et al. 2017). According to Eq. 5, the maximum heat loss rate of the MTHX is estimated to be around four times the STHX’s heat losses under the same operating condition.

## Experimental results and discussions

### Comparative analysis between the STHX and MTHX

**Fig. 5:**
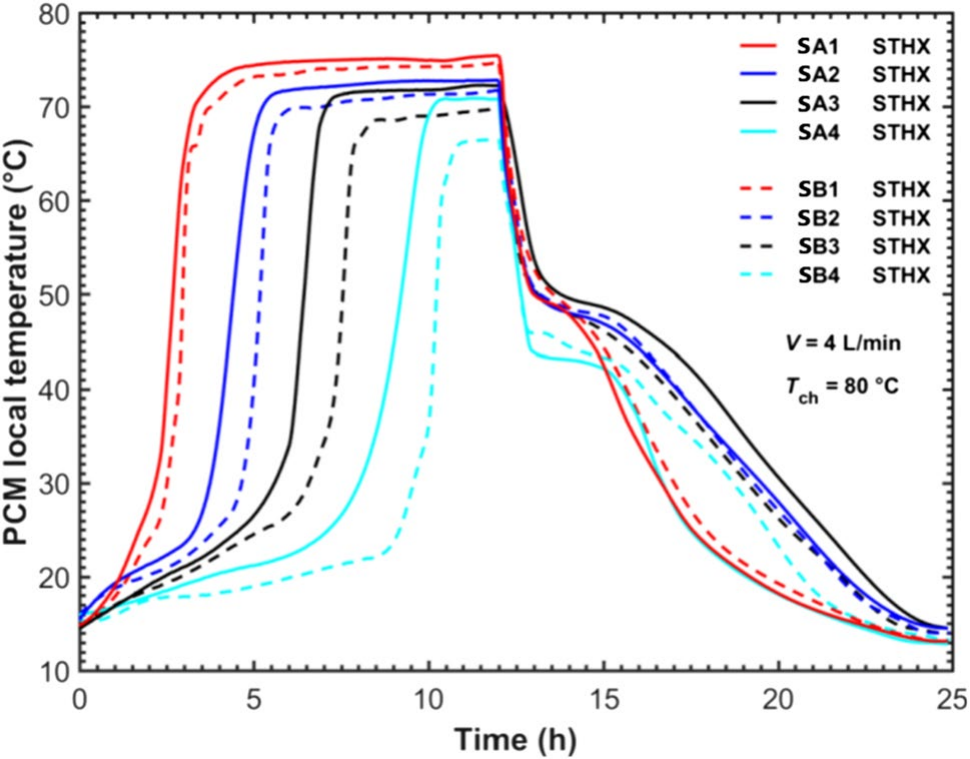
PCM local temperature evolutions in the STHX

Figure 5 plots the evolution of PCM temperatures recorded at positions SA and SB (see Fig. 2a) during a complete charging and discharging process in the STHX. The charging process was operated with the HTF inlet temperature of 80 °C and a flow rate of 4 L/min, immediately followed by the discharging process with the inlet HTF temperature of 15 °C and the same flow rate. For positions SA and SB, it was noticeable that the PCM temperature at a higher level rose faster and began to stabilize sooner than that at a lower level. This indicated natural convection played an important role during the melting process. Due to the natural convection, the hot liquid PCM moved upwards and accelerated the melting process in the upper part of the LHTES system by enhancing the PCM heat transfer. This justified that the natural convection dominated the heat transfer in the melting process as reported in the literature (Seddegh et al. 2017). At the same level, position SB recorded lower temperatures than position SA. This was because position SB was further away from the HTF tube in comparison to position SA. It took much longer time for the heat to be transferred from the HTF to PCM at point SB. Also, the PCMs at point B were close to the shell surface, and heat loss to the environment also affected the temperature at this point. On the other hand, with heat conduction dominating heat transfer throughout the solidification, there was no significant difference in the pattern of PCM temperature evolution at the same PCM level. The STHX was observed to experience around 25 h of a total charging and discharging duration at the charging temperature of 80 °C.

**Fig. 6:**
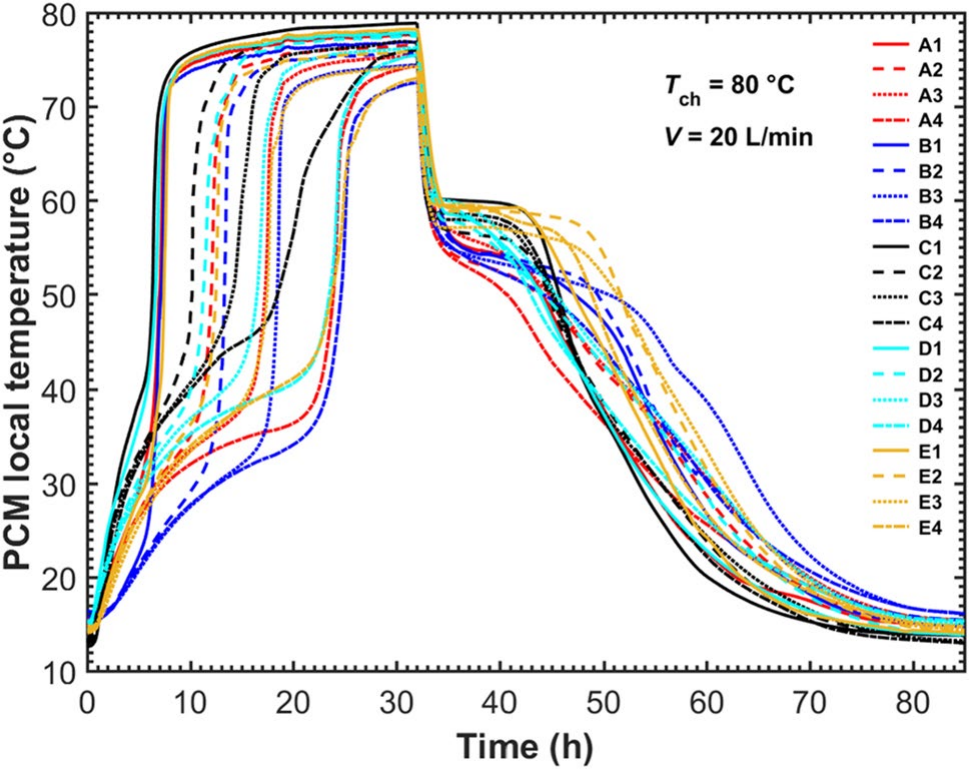
PCM local temperature evolution in the five-tube HX

Figure 6 shows PCM temperatures recorded at positions A, B, C, D, and E within the five-tube HX LHTES system. The complete charging and discharging process was operated at the same charging and discharging temperatures as those in the STHX presented in Fig. 5. The total HTF flow rate was 20 L/min on the five-tube HX so the HTF flow in each tube was 4 L/min same as that in the STHX. It was observed from Fig. 6 that during the charging, PCMs at a higher level melted faster, and their temperature stabilized sooner, which was similar to that reported in the STHX. Besides, as noted in the “Experimental rig and procedure” section, position B was the most faraway from HTF tubes and in turn, could monitor if the PCMs at this level were fully melted or solidified. The temperature recordings at position B1 indicated that a whole liquid PCM pool emerged at the PCM top region at the charging time *t* = 6 h, formed from the separate melted PCM layers along HTF tubes. Then, the liquid PCM volume gradually extended downwards until the lowest level sensor B4 surpassed 61 °C (PCM liquidus temperature) at around 26 h of charging when the upper-level temperature sensors B1, B2, and B3 had all stabilized. Overall, the evolution pattern of the liquid PCM volume inside the MTHX was similar to that in the STHX unit. The complete charging and discharging process of the five-tube MTHX lasted around 85 h. The cycle charging and discharging rate per tube in the MTHX was 0.08 kg/h per tube which was lower than 0.11 kg/h per tube. This was because more than half of PCMs were located outside of the virtual optimal tube boundary in the MTHX leading to a longer time for melting and solidification. This implied that more tubes were required to achieve the same cycle charging and discharging rate in the MTHX. It also indicated that the numerical model by simplifying the multitube model into the virtual single tube one used in the literature (Trp et al. 2006; Tehrani et al. 2016; Pirasaci and Goswami 2016; Fang et al. 2018) might not be able to accurately capture the thermal characteristics of the MTHX. A full mathematical model might be necessary.

**Fig. 7:**
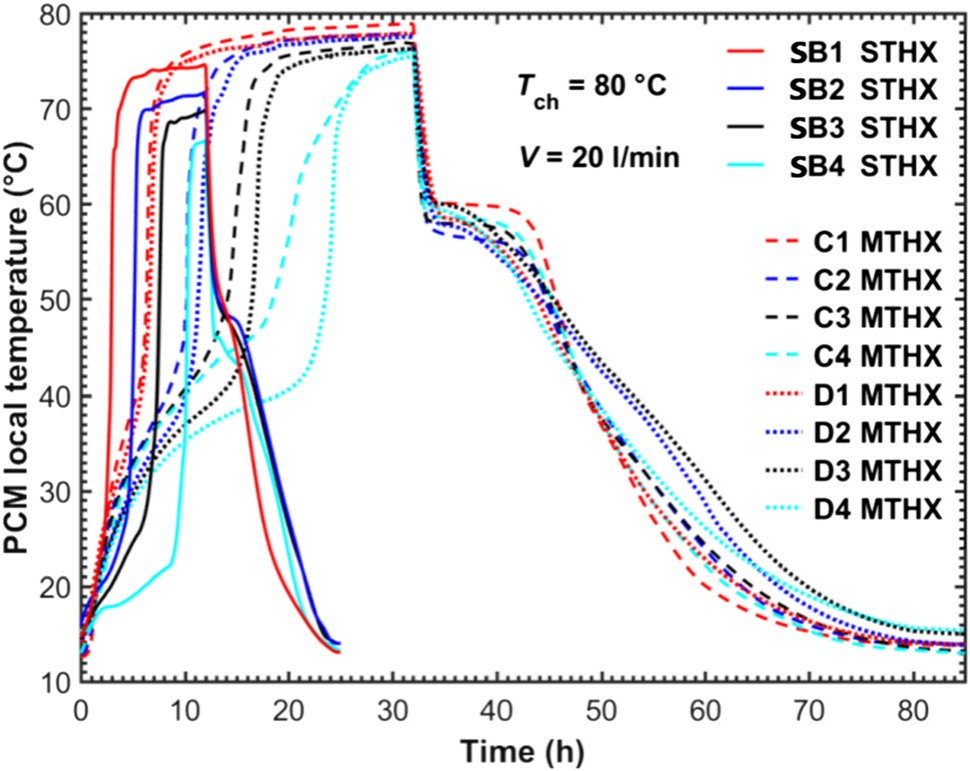
Comparison of local temperatures between the STHX and five-tube MTHX

To verify the common numerical modelling and to simplify the multitube model into a single-tube one (Trp et al. 2006; Tehrani et al. 2016; Pirasaci and Goswami 2016; Fang et al. 2018), Fig. 7 compares the temperature evolutions between position SB in the STHX and positions C and D in the five-tube MTHX. According to Fig. 2b and c, position SB inside the STHX was mounted 45 mm away from the HTF tube central axis, while positions C and D inside the MTHX were placed at the same distance from the central- and outer-tube axes respectively. Positions C and D of the MTHX could be used to reflect the thermal characteristics of the central and outer virtual cylindrical boundaries. Therefore, position SB of the STHX and positions C and D of the five-tube MTHX are comparable to reveal the deviation in thermal behavior between the STHX and the virtual cylindrical boundaries in the MTHX. It was observed from Fig. 7 that position C or D at each level in the MTHX melted much slower than position SB in the STHX. This was mainly because the MTHX contained more than twelve times the amount of PCM but had only five times the heat transfer area configured compared to the STHX. In particular, more than half of the PCMs were located outside the optimal virtual tube boundary. Moreover, positions C and D in the MTHX were observed to climb to higher temperatures at the end of charging. This could be explained by the longer charging time as it took much longer time for the energy to be transferred to PCMs allocated at the place close to the shell’s inner surface.

Within the MTHX, the results at positions C (located at central) and D (located at outer) in Fig. 7 showed a noticeable difference in thermal performance between central and outer HTF tubes. It was seen that at the same level, position C melted faster and then recorded a higher stable temperature at the end of charging. The difference can be attributed to two factors: (1) apart from the central tube, two adjacent outer tubes also affected the PCM melting at position C and (2) position D suffered more heat loss to the surroundings.

**Fig. 8:**
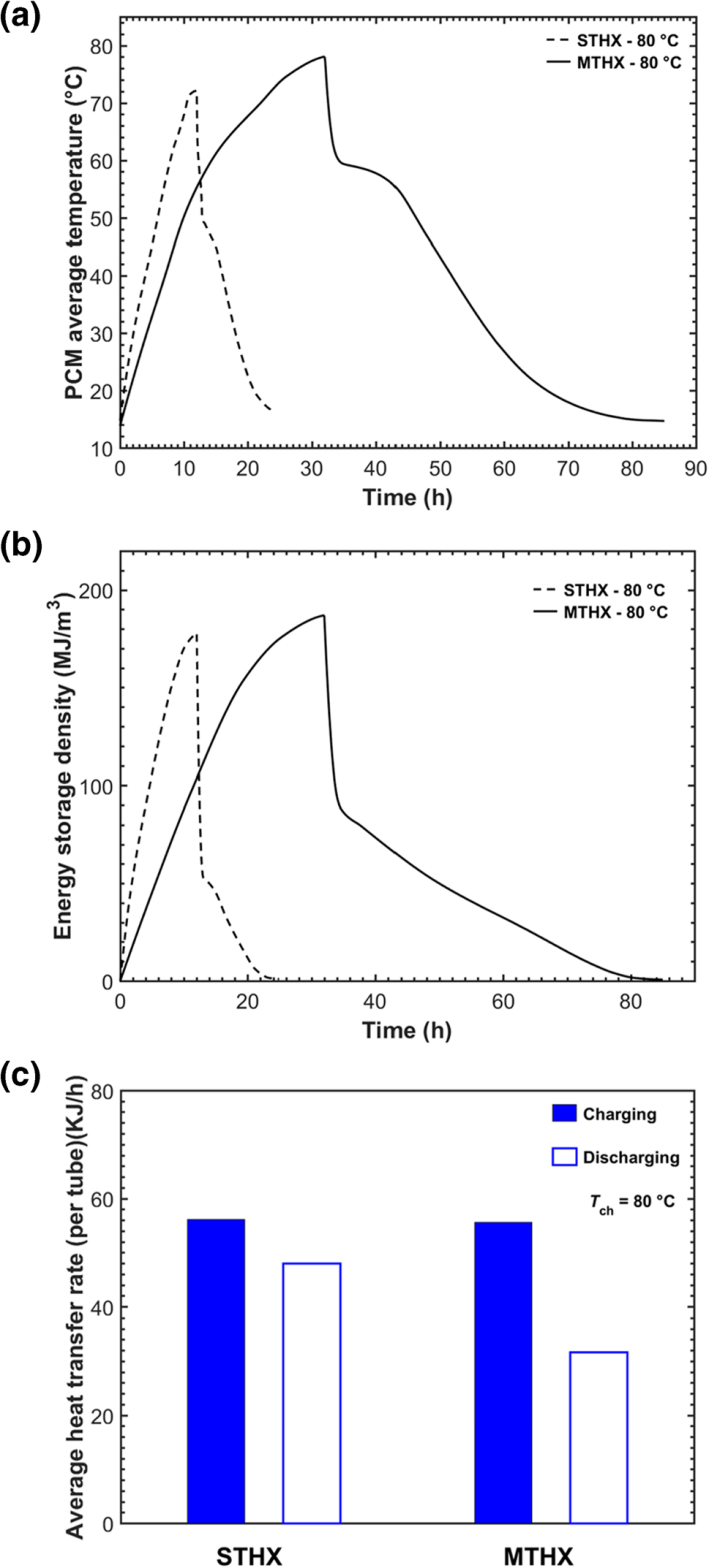
Comparisons of thermal responses between the STHX and the five-tube MTHX in terms of **a** PCM average temperature, **b** energy storage density, and **c** average heat storage/release rate

Figure 8 compares the overall performance between the STHX and the five-tube MTHX in terms of the average PCM temperature, energy storage density, and average heat exchange rate per tube. It was shown in Fig. 8a that the MTHX attained a higher maximum average temperature of 77 °C compared to 72 °C by the STHX at the same HTF inlet temperature of 80 °C. During melting in the MTHX, multiple liquid PCM cells initially developed along the tubes and then merged into a whole pool at the top PCM region due to natural convection. The extended solid–liquid fronts and multiple heat sources contributed to more effective natural convection in the MTHX. Moreover, the MTHX experienced a much longer charging time (32 h), leading the melted PCM to be continually heated and thus resulting in a higher maximum average temperature at the end of charging. Overall, Fig. 8a showed that the STHX were around 62.5% faster in charging (*t*_ch_, 12 vs 32 h) and 74% faster in discharging (*t*_dis_, 14 vs 53 h) than the MTHX. This could be explained by the much larger ratio of PCM mass (~ 12, MTHX to STHX) compared to the ratio of heat transfer surface area (5, MTHX to STHX) between the two HXs.

Figure 8b compares the energy storage density (*Q*/*V*) between the STHX and MTHX. The cumulative energy stored *Q* is calculated for each heat exchanger using Eq. 4. The maximum energy storage density in the MTHX is slightly higher than that in the STHX, due to the difference in sensible heat over a much longer charging time.

Figure 8c compares the average heat transfer rate per tube within the STHX and MTHX. The average heat transfer rate is calculated as *Q*/(*t* × n), where *t* is the total charging/discharging time, and *n* is the tube number. During charging, the average heat storage rate differs slightly between the two heat exchangers. However, it should be noted that the average heat storage rate per tube in the MTHX is influenced by more complex factors, including tube distribution, tube number, and the total PCM mass. On the other hand, the average discharging rate per tube in the MTHX is significantly lower compared to the STHX. This could be explained by the much longer discharging time and the evolution of PCM solid–liquid interfaces in the MTHX. At the later stage of discharging in the MTHX, the PCM solid–liquid interfaces move extremely slowly due to a continual increase in the thermal resistance between the HTF tube and liquid PCM, which greatly reduces the discharging rate.

In this study, a large portion of PCM was excluded from the virtual cylindrical boundaries formulated in the MTHX due to the specific tube number and the tube spacing intending to agree with an optimal shell-to-tube radius ratio. This factor largely leads to the significant differences in charging and discharging processes between the STHX and the MTHX. However, even with many more tubes appropriately arranged within the MTHX, almost half of PCM will be inevitably excluded from virtual cylindrical boundaries. And there are still deviations both between the STHX to the central or outer virtual cylindrical boundary in the MTHX and between the two HXs in the overall thermal response.

Besides, as detailed in the “Heat loss analysis” section, the total heat loss from an STHX or MTHX was not only affected by its outside surface area but also coupled with the dynamic temperature differences between PCM and the environment. There is a difference in thermal performance between the outer and inner tubes in the MTHX as revealed in Fig. 7, which cannot be addressed by a simplified single-tube model. In summary, the comparative study of the two HXs clearly demonstrated that there remained a need for a comprehensive investigation of the vertical multitube LHS system, which must rely on an experimental study or three-dimensional numerical model. This is consistent with the findings in the literature (Agyenim et al. 2010; Joybari et al. 2019; Pizzolato et al. 2019).

### Effect of tube number in the MTHXs

To study the effect of varying the inner tube number within the MTHXs, two- and four-inner-tube MTHX were implemented with the same storage enclosure size and tube specifications as the five-tube MTHX. As the inner tube number varied from five to four or two, the thermocouple positions were placed at the same positions, and the total PCM amount was held constant. The centrosymmetric placement of inner tubes in the three cases of the MTHX, as shown in Figs. 2c and 4, is promising for the vertical MTHX, enabling a relatively uniform PCM melting and solidifying. The constant total flow rate of 20 L/min was distributed among the inner tubes during this series of tests along with the HTF charging temperature of 80 °C and a default discharging temperature of 15 °C.

#### Local temperature comparisons at central and outer tubes

**Fig. 9:**
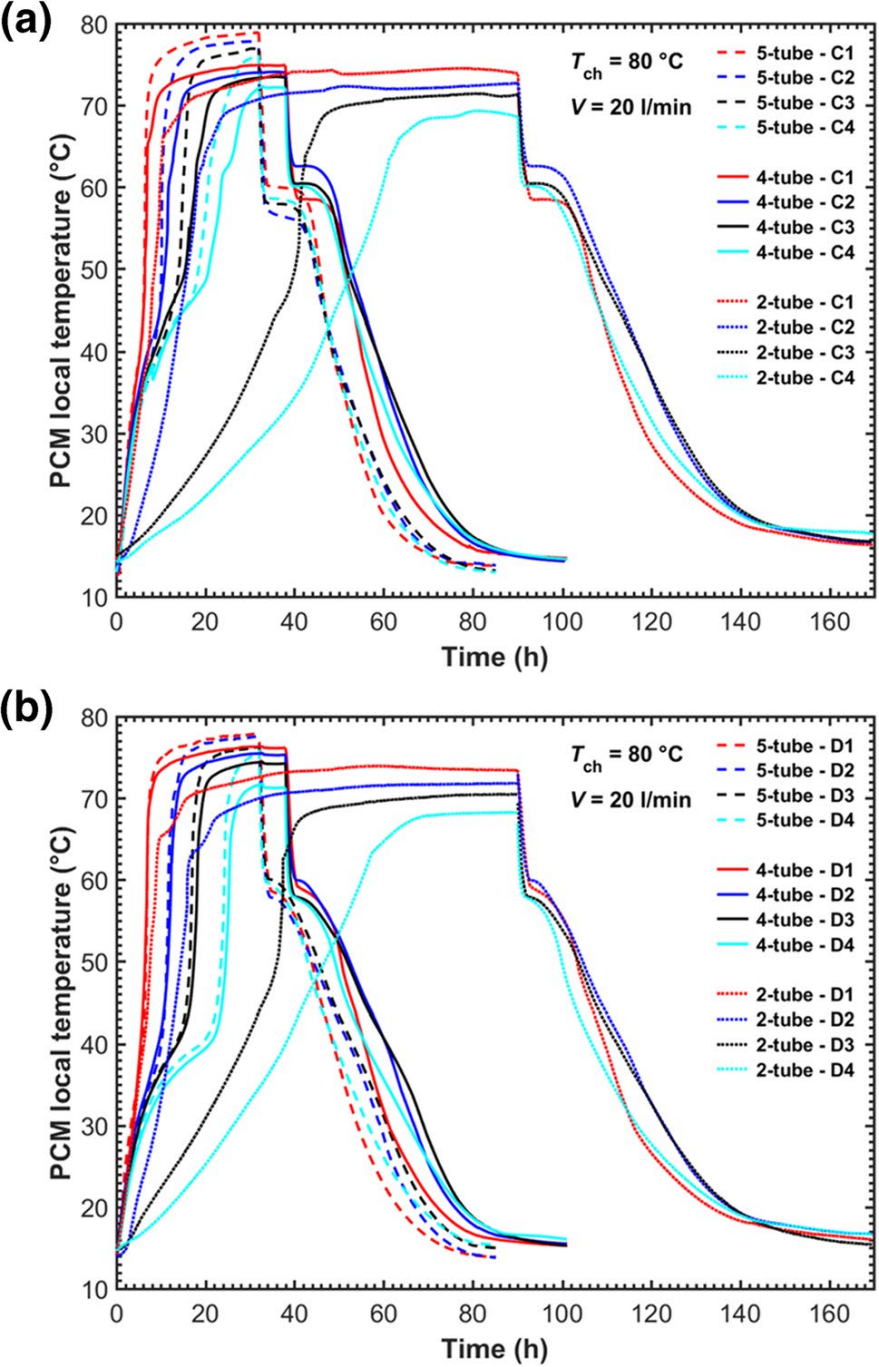
Comparisons of the PCM local temperature at positions C (**a**) and D (**b**) under different tube numbers

Figure 9a and b compares the thermocouple readings at central (position C) and outer (position D) positions under the different inner tube numbers in the MTHXs. It was seen that during the charging, position C within the five-tube case melted faster and attained a higher maximum temperature, compared to the same position in the case with two or four tubes. For instance, the highest level sensor C1 within the five-tube case attained the maximum temperature of 79 °C compared to 72 °C at the same position within the two-tube case. This indicates the central tube within the five-tube case resulted in a higher heat transfer rate and more effective natural convection, hence the higher maximum temperature at position C.

Figure 9b shows the five-tube case also melted faster and recorded higher temperature at the outer region of PCM (position D) than the cases with fewer tube numbers. The result proved that the addition in tube number helped enhance heat transfer to the remote region of PCM. For the discharging performance, it was seen in Fig. 9a and b that the temperatures at positions C and D within the five-tube case both dropped faster in temperature, resulting in a shorter discharging process compared to those of the four- and two-tube cases. These are consistent with the common practice that more heat transfer areas with more tubes could enhance the heat transfer in the PCM, and hence the multitube design is a useful and effective enhancement technique to improve the thermal performance of LHTES systems.

#### Overall performance comparison

**Fig. 10:**
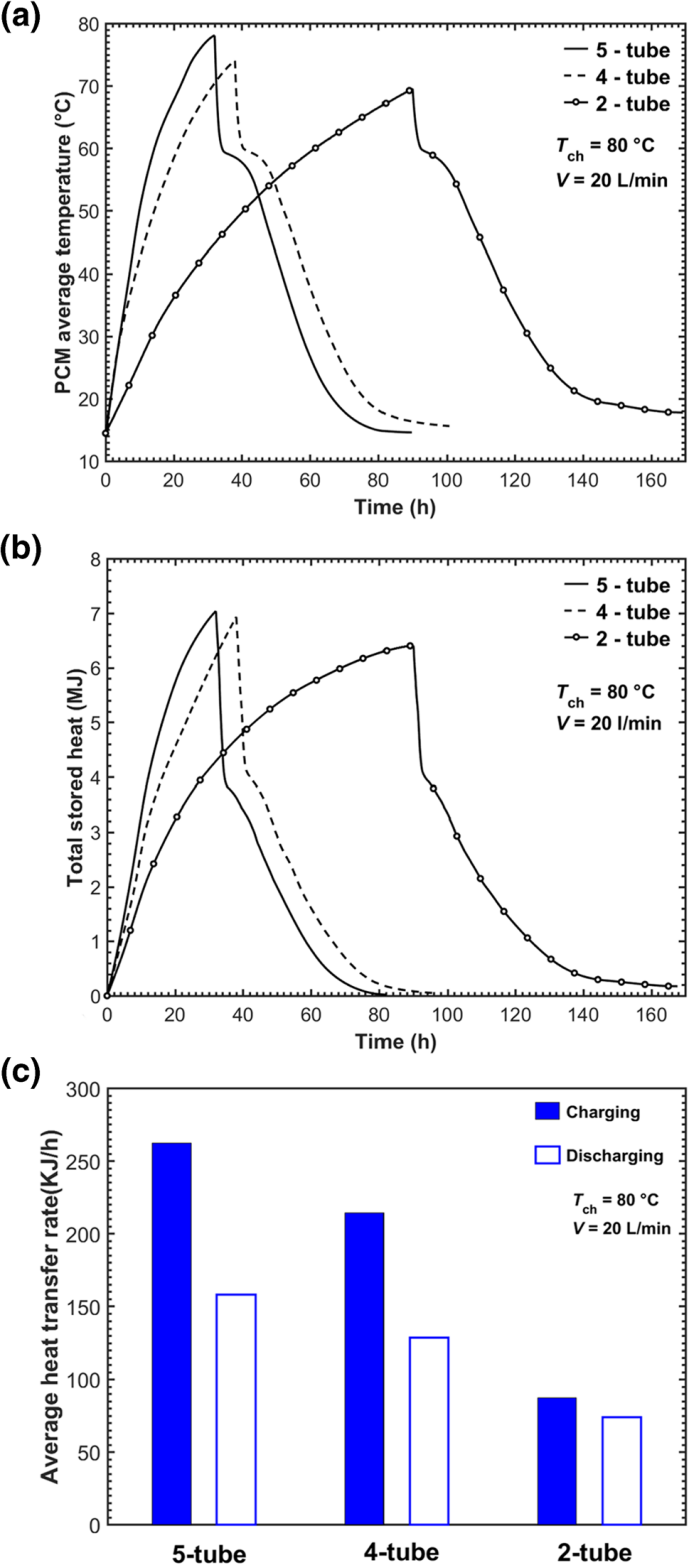
**a** The average temperature, **b** total stored heat, and **c** average heat exchange rate in the MTHXs with different tube numbers

Figure 10a and b shows the overall performance of the MTHXs with different tube numbers in terms of the average PCM temperature and total stored energy. Figure 10a shows that the maximum average temperature was higher with an addition in the tube number, similar to the pattern revealed by the PCM local temperature. Figure 10b indicates that the maximum total stored heat by the five-tube case was 1.5% and 10% higher than those by the four- and two-tube cases, respectively. Figure 10c compares the average heat transfer rate within the MTHXs, which is calculated as *Q*/*t* where *t* is the total charging/discharging time. The tube number significantly affected the charging and discharging rates. The charging and discharging rates of the five-tube case were 202% and 115%, respectively, higher compared to the two-tube case.

In the vertical MTHX, PCM natural convection flow takes place between the HTF tubes. Figures 9 and 10 demonstrated that more tubes added to a vertical MTHX promoted both charging and discharging performance without inhibiting the natural convection. This was different from the findings regarding the horizontal MTHX in which tubes placed at the upper PCM region may interfere with natural convection thus reducing the charging rate (Kousha et al. 2019). However, adding more tubes is not always desirable because it would lead to the PCM amount loss and the cost increase in the LHTES unit. An optimal inner tube design needs to ensure the benefit in heat transfer from the tubes added is well worth the costs.

### Effects of HTF operating parameters in the five-tube MTHX

#### HTF inlet temperature

**Fig. 11:**
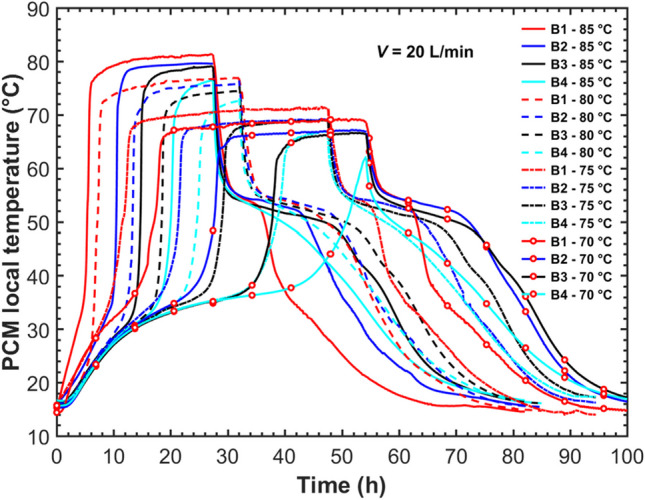
Evolution of temperatures at position B in the five-tube MTHX under different charging temperatures

To analyze the effect of HTF inlet temperatures, the charging temperatures of 70 °C, 75 °C, 80 °C, and 85 °C combined with a constant discharging temperature of 15 °C were performed on the five-tube MTHX, while the HTF flow rate was maintained at 20 L/min. Figure 11 shows PCM temperature evolutions at position B during the complete charging and discharging process. As already noted, position B could monitor the melting and solidification status of the PCMs at the corresponding level. It was obtained from Fig. 11 that the HTF inlet temperature affected the charging time significantly but showed a relatively slight influence on the discharging time. The charging time was reduced by 12%, 32%, and 14% for the HTF inlet temperature to increase from 70 °C to 75 °C, from 75 °C to 80 °C, and from 80 °C to 85 °C, respectively. At the same time, the corresponding increasing rates in the discharging time were 2%, 11%, and 3%. This is associated with the heat transfer mechanism in the PCM solidification process. The PCM temperature fell fast at the initial discharging stage (*t*_dis_ < 2 h), and then the thermal resistance between the HTF and liquid PCM increased considerably with PCM solidifying on the tube surface. This led to the difference in the PCM initial temperatures at discharging playing a less important role in the whole discharging duration. In other words, the total charging and discharging duration mainly depended on the charging section under different HTF charging temperatures.

**Fig. 12:**
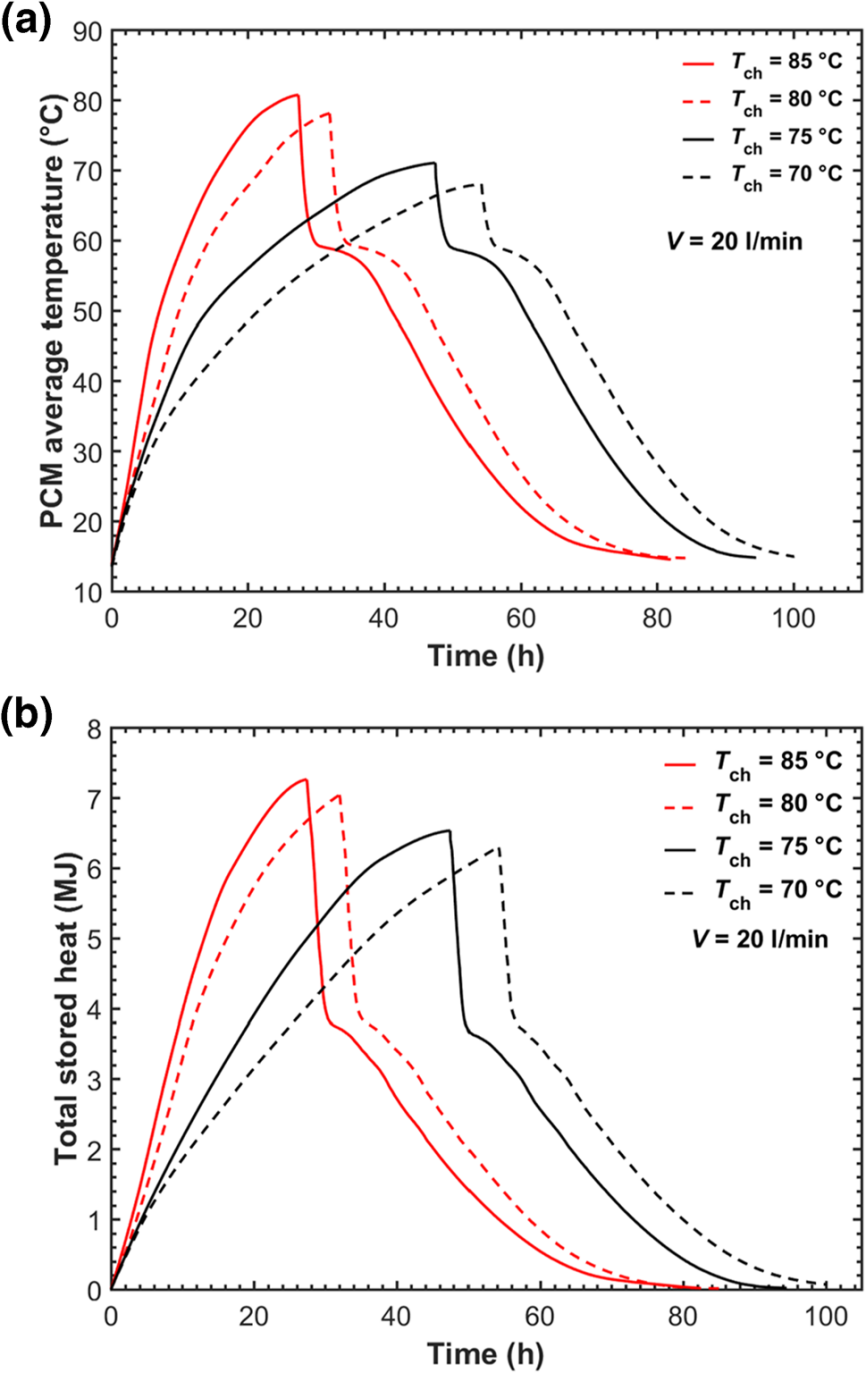
Comparisons of PCM average temperature (**a**) and the total stored energy (**b**) in the five-tube MTHX under different charging temperatures

Figure 12a and b shows the average PCM temperature and the total stored heat to evaluate the overall performance with the four HTF inlet temperatures operated. It was seen that the HTF temperature exhibited a noticeable influence on the average PCM temperature and the total amount of heat stored. As the HTF temperature increased from 75 °C to 80 °C, the maximum average temperature was around 7 °C higher, and the maximum stored heat rose by 8% with the charging time reduced by 32%. The improvement in the total stored energy with the different HTF temperatures operated could be attributed to the deviation in sensible heat stored as the PCM melted at the end of charging.

#### Effect of the HTF flow rate

To investigate the effect of the HTF flow rate on the performance of the MTHX system, another flow rate of 5 L/min was performed on the five-tube MTHX at a charging temperature of 85 °C. Both flow rates of 5 and 20 L/min are distributed equally among the five inner tubes, resulting in a turbulent flow with Reynolds numbers being 3500 and 14,000, respectively within each tube (see Table 2). The comparisons between the flow rates are presented by the temperature evolutions at individual thermocouples (positions C and D) and the overall thermal performance in terms of the PCM average temperature and the total stored heat.

**Fig. 13:**
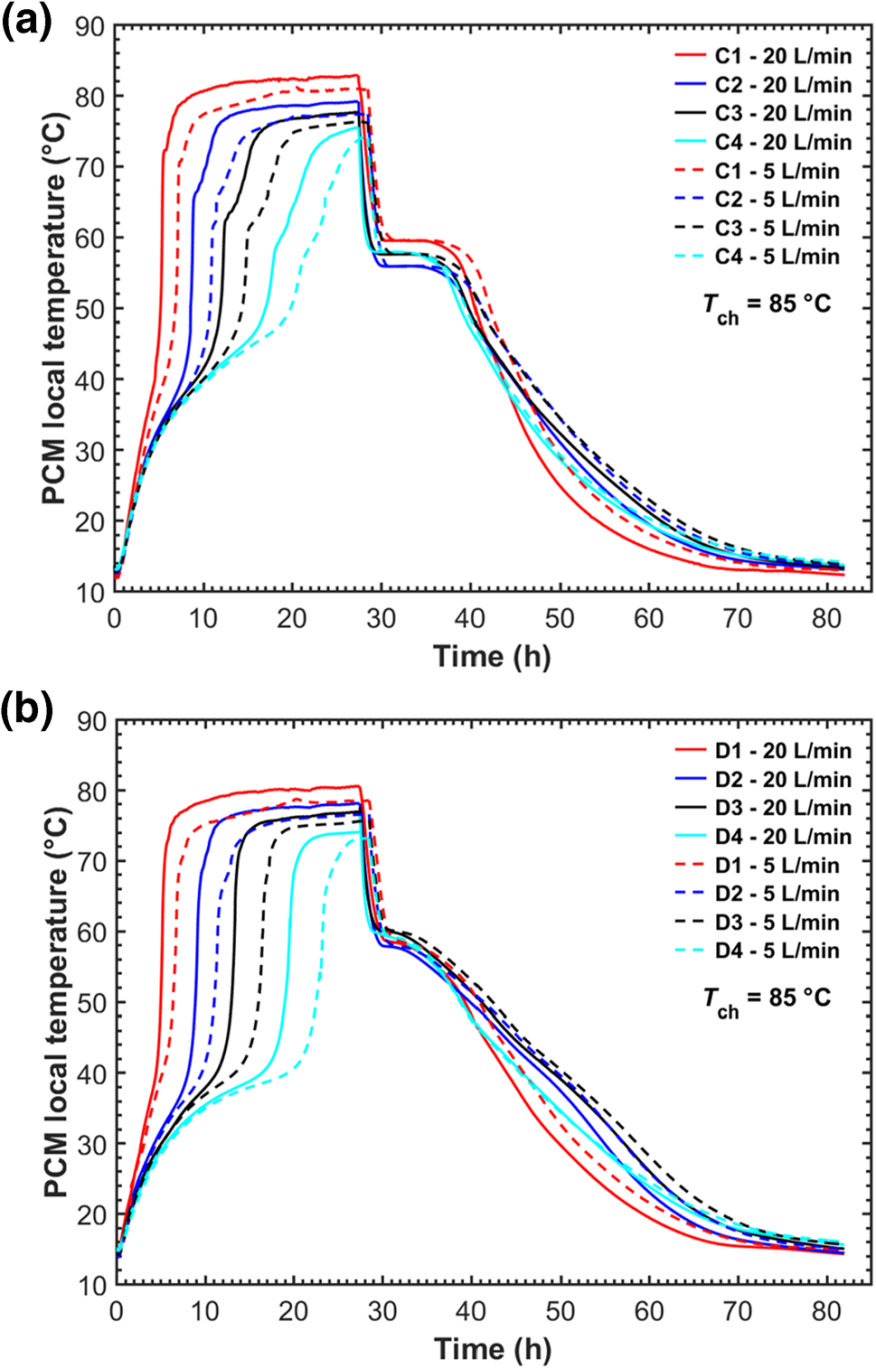
Temperature evolutions at positions C (**a**) and D (**b**) under two HTF flow rates within the five-tube MTHX

Figure 13a and b shows the temperature evolutions under two HTF flow rates at positions C and D, which were located at the same radial distance from the central and outer tubes, respectively. It was found that the effect of the HTF flow rate on the system was different between the charging and discharging processes based on the temperatures recorded by the individual thermocouples. A higher flow rate resulted in higher temperatures during the charging process while showing a negligible effect on the discharging process. The PCM temperatures were independent of the HTF flow rate at the initial charging stage (*t*_ch_ < 1.5 h) and then increased faster at a higher flow rate. At the end of charging, the temperatures climbed to higher values at the flow rate of 20 L/min, indicating an improvement in the heat transfer. Comparatively, during discharging, temperature plots at both positions C and D under the two HTF flow rates nearly overlapped following a short duration of discharging (~ 1 h). Such different effects on the charging and discharging could be explained by the different dominant heat transfer mechanisms over the two cycles. In the melting process, once liquid PCM layers were formed around the tubes, natural convection dominated heat transfer; hence, the enhancement effect by a higher HTF flow rate became pronounced. During the solidification process controlled by heat conduction, the higher thermal resistance of the solidified PCM layer on the tube inhibited heat transfer between the liquid PCM and the tube, resulting in a marginal impact by a higher HTF flow rate on the overall heat transfer rate. This finding is also in agreement with the results of the experimental studies (Murray and Groulx 2014b; Kabbara et al. 2016) and the numerical study (Shen et al. 2019b).

**Fig. 14:**
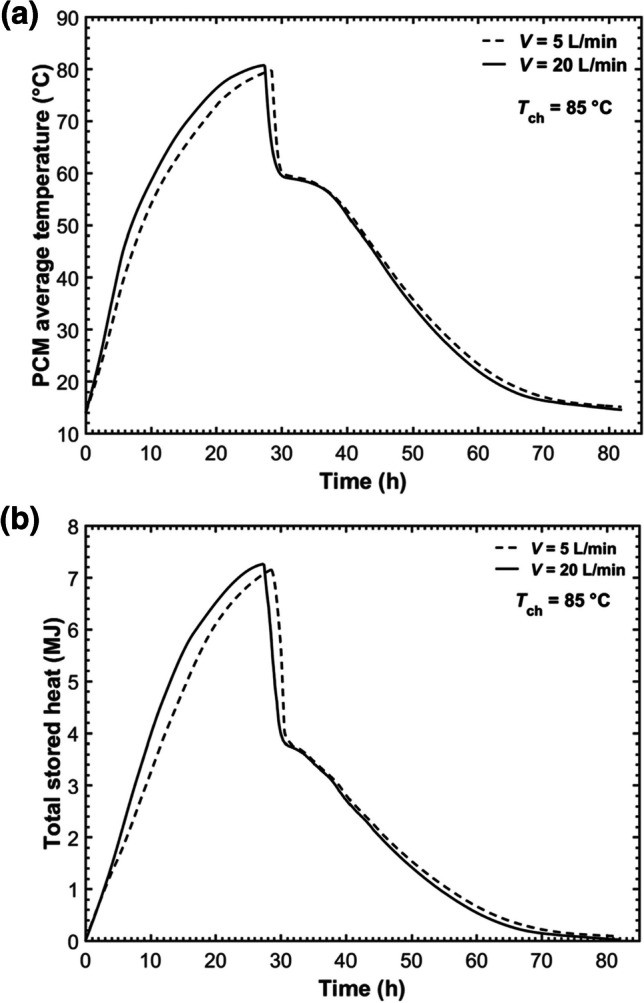
Comparisons of **a** PCM average temperature and **b** the total stored heat under different flow rates within the five-tube MTHX

The comparisons of PCM average temperature and the total stored energy under the two flow rates within the five-tube MTHX are presented in Fig. 14. Increasing the HTF flow rate from 5 to 20 L/min exhibited a slight increase in average temperature during the charging process. A higher flow rate also enhanced the total stored energy during charging and led to a higher maximum stored energy at the end of charging. The charging time decreased slightly under a high flow rate, while the total charging and discharging time was almost the same between the two HTF flow rates. This is because the discharging time was much longer than the charging time for both cases, and the HTF flow rate has a negligible impact on the discharging process.

## Conclusions

This work experimentally investigated the charging and discharging characteristics in the vertical STHX and MTHXs. Firstly, this work verified a simplified numerical solution commonly used in the literature that formulated a virtual cylindrical PCM domain along each tube, converting a three-dimensional multitube problem to a two-dimensional single-tube model. Secondly, the impact of inner tube number in the MTHX on thermal performance was investigated in terms of PCM local temperatures, PCM average temperature, and the total stored heat. Finally, the effects of HTF charging temperatures and flow rates were examined on the five-tube MTHX. Some specific conclusions are listed below:

1. The temperatures in the STHX were substantially different from those measured at the same position from the HTF tube in the central and outer virtual cylindrical domains in the five-tube MTHX at the same operating conditions. The overall thermal behavior between the STHX and MTHX also showed significant differences. The deviations were due to the following facts: (i) a large part of PCMs was inevitably excluded from virtual cylindrical boundaries formulated in the MTHX, and (ii) the total heat loss from the two LHTES units was different. In the MTHX, the heat transfer to the virtual cylindrical boundary was influenced by both its adjacent tubes and its central or outer location. There is no linear or simple correlation in heat transfer between the STHX and the MTHX.
2. The centrosymmetric placement of inner tubes enabled more uniform melting and solidifying in a vertical MTHX. The larger tube number considerably improved both charging and discharging rates. The maximum temperature (79 °C) was recorded in a five-tube system, and it was higher than those in a four-tube (74 °C) or two-tube unit (72 °C), indicating more tubes resulted in more effective natural convection in a vertical cylindrical system.
3. A higher HTF charging temperature significantly improved the charging rate in the MTHX, while the discharging duration is less sensitive to the HTF charging temperature. The corresponding increasing rates in the discharging time were relatively lower (≤ 11%) for each 5 °C increase in the charging temperature. Moreover, the HTF flow rate exhibited a more pronounced effect on the charging than on the discharging.

## Nomenclature

**Table:** 

*C*_*p*_	Specific heat	(J/kg∙K)
*d*	Tube outer diameter	(mm)
*D*	Shell inner diameter	(mm)
*g*	Acceleration of gravity	(m/s^2^)
*H*	Unit height	(mm)
*L*	Latent heat	(J/kg)
*m*	Mass of PCM	(kg)
n	Tube number	(-)
*Q*	Stored energy	(MJ)
Re	Reynolds number	(-)
*t*	Time	(s)
*T*	Temperature	(°C)
*U*	Overall heat transfer coefficient	(W/m^2^ ∙K)
*v*	PCM volume at *T* = 15 °C	m^3^
*V*	HTF volume flow rate	(L/min)
*x*	Radial coordinate	(mm)
*z*	Axial coordinate	(mm)
*Greek letters*		
*ϕ*	Liquid fraction	(-)
*ω*	Weighting factor	(-)
*ρ*	Density	(kg/m^3^)
*Subscripts*		
ch	Charging	
dis	Discharging	
i	Element	
ini	Initial	
liquidus	Liquid phase	
loss	Heat loss	
solidus	Solid phase	

## Data Availability

All data analyzed in the study are included in this article.

## Abbreviations

*HTF*: Heat transfer fluid
*HX*: Heat exchanger
*LHTES*: Latent heat thermal energy storage
*MTHX*: Multiple-tube heat exchanger
*PCM*: Phase change material
*STHX*: Single-tube heat exchanger
